# Allen key completely in male urethra: a case report

**DOI:** 10.4076/1757-1626-2-7408

**Published:** 2009-07-20

**Authors:** Michael Mitterberger, Reinhard Peschel, Ferdinand Frauscher, Germar M Pinggera

**Affiliations:** 1 Department of Urology, Medical University InnsbruckAnichstrasse 35, A-6020 InnsbruckAustria; 2 Department of Radiology, Medical University InnsbruckAnichstrasse 35, A-6020 InnsbruckAustria

## Abstract

**Introduction:**

Various cases of self-inflicted foreign bodies in the male urethra have been reported. Most of them are associated with autoerotic stimulation, psychiatric disorders or intoxication.

**Case presentation:**

We report the first case of a patient who put an Allen key completely in his urethra. The patient presented with dysuria, haematuria and penile pain.

**Conclusion:**

A self-inflicted urethral foreign body is a rare situation. Endoscopic removal is the recommended first-line treatment and if unsuccessful, open procedures may be necessary.

## Introduction

Various cases of self-inflicted foreign bodies in the male urethra have been reported [1]. Most of them are associated with autoerotic stimulation, psychiatric disorders or intoxication [2]. The patients present with dysuria, haematuria, urinary retention, penile pain or swelling [3]. We report about a patient who put an Allen key completely in his urethra.

## Case presentation

**Figure 1.**
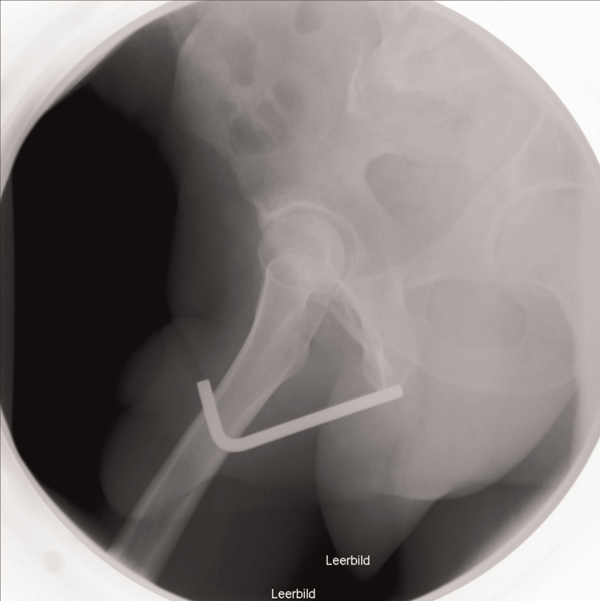
X-ray of the pelvis showing the Allen key completely in the anterior urethra.

**Figure 2.**
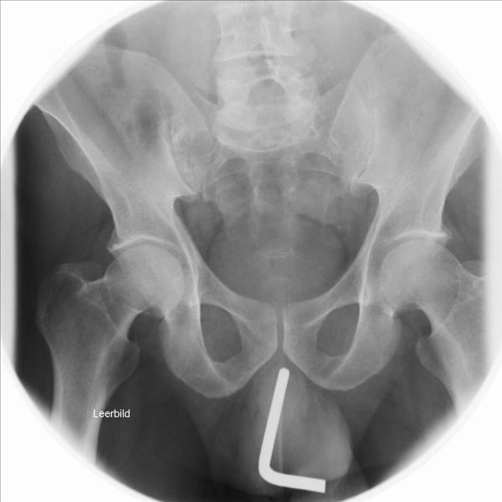
X-ray of the pelvis showing the Allen key in the urethra.

**Figure 3.**
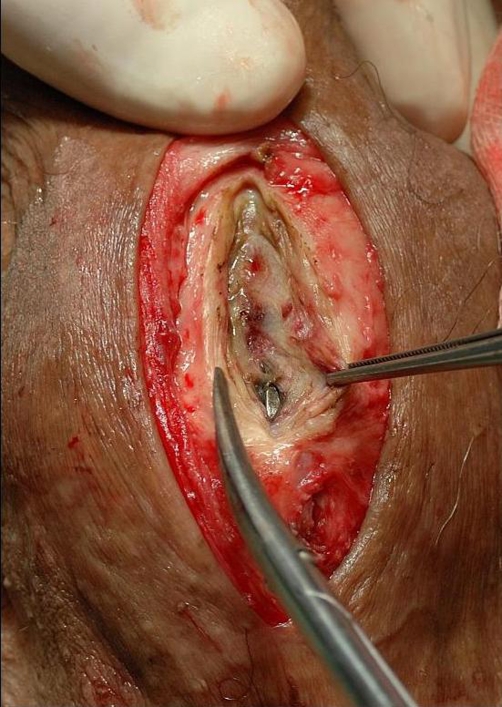
External urethrotomy of the anterior urethra showing the metallic object in the urethra.

**Figure 4.**
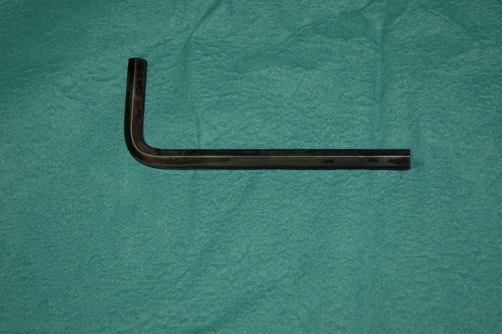
Allen key removed from urethra.

A 51-year-old Caucasian male presented to the emergency room and stated that he has an Allen key in his urethra since two days. The patient could void and had bloody urethral discharge. Further he had made several unsuccessful attempts to remove it. His physical examination showed a normal urethral meatus and a palpable foreign body within the penis. An x-ray of pelvis demonstrated a 9 cm long metallic object in the anterior urethra, corresponding an Allen key (Figures 1 and 2). The patient was not married and his socioeconomic status was of upper class. It was the first time he had ever self-inflicted a foreign body in his urethra and he had no history of drug addiction or psychiatric illness. After giving his formal consent, the patient was taken to the operating room. Under general anesthesia and fluoroscopic control, an attempt was made to pull out the foreign body with a 22Fr cystoscope. This was unsuccessful, due to lack of space. Then an external urethrotomy of the anterior urethra was performed, the Allen key was removed and an 18F urethral catheter was placed (Figures 3 and 4). The patient was discharged two days later with the catheter. After three weeks under oral antibiotic therapy a voiding cystourethrogram (which detected no abnormalities) was performed and the patient was discharged again. Weeks later the patient presented himself again showing a long penile stricture. Therefore a urethral reconstruction had to be performed.

## Discussion

Various self-inflicted foreign bodies have been reported in the male urethra [1]. Objects like nuts, pens, wire and others have been described. The clinical presentation of the patient may vary from asymptomatic to swelling of external genitalia, dysuria, poor urinary stream, urethral discharge or urinary tract infection [3]. The main reason for foreign body self-insertion is of sexual nature, like masturbation or sexual gratification [2]. Also a mental illness or drug intoxication may be the reason. Most of the patients feel guilty and ashamed and do not seek medical help until they experience severe voiding problems. In our case, the patient underwent several unsuccessful attempts to remove the foreign body and came with a delay of two days.

Interesting underlying psychiatric and psychoanalytic theories have been postulated [4]. However, the psychiatric evaluation is controversial as many of these patients are psychologically normal. Also in our case, the psychiatrist evaluation was normal and the patient revealed no signs of depression or impulsive behavior.

The primary goal of the treatment should be removal of the foreign body with little damage to the urethra as possible. Further various methods of foreign body removal have been described, depending on the form, size and mobility of the body and its location in the urethra [5]. Endoscopic therapy is the standard care, but in cases where the foreign body cannot be removed open surgery becomes necessary. In our case, due to the ninety degree angle of the Allen key, endoscopic removal was unsuccessful and an external urethrotomy had to be performed.

Known long term complications of self-inflicted urethral foreign bodies are urethral stricture or diverticulum, incontinence or erectile dysfunction. Further the rationale for the behaviour should be investigated to prevent recurrence [1].

## Conclusion

A self-inflicted urethral foreign body is a rare situation. Endoscopic removal is the recommended first-line treatment and if unsuccessful, open procedures may be necessary.
